# Receptor for Advanced Glycation End Products Antagonism Blunts Kidney Damage in Transgenic Townes Sickle Mice

**DOI:** 10.3389/fphys.2019.00880

**Published:** 2019-07-23

**Authors:** Emmanuelle Charrin, Camille Faes, Amandine Sotiaux, Sarah Skinner, Vincent Pialoux, Philippe Joly, Philippe Connes, Cyril Martin

**Affiliations:** ^1^ Interuniversity Laboratory of Human Movement Biology, University Claude Bernard Lyon 1, University of Lyon, Lyon, France; ^2^ Laboratory of Excellence “GR-Ex”, Paris, France; ^3^ Institut Universitaire de France, Paris, France; ^4^ Groupement Hospitalier Est, UF “Biochimie des Pathologies érythrocytaires” Centre de Biologie Est, CHU de Lyon, Lyon, France

**Keywords:** sickle cell disease, RAGEs, kidney, Townes mice, oxidative stress, inflammation

## Abstract

A large proportion of adult patients with sickle cell disease (SCD) develops kidney disease and is at a high risk of mortality. The contribution of advanced glycation end products and their receptor (AGE/RAGE) axis has been established in the pathogenesis of multiple kidney diseases. The aim of the present study was to determine the implication of RAGE in the development of SCD-related kidney complications in a mouse model of SCD, as this has never been investigated. 8-week-old AA (normal) and SS (homozygous SCD) Townes mice were treated with a specific RAGE antagonist (RAP) or vehicle (NaCl). After 3 weeks of treatment, red blood cell count, hematocrit, and hemoglobin levels were significantly higher in RAP-treated SS mice. Reticulocyte count and sickle cell count were reduced in RAP-SS compared to their NaCl-treated littermates. The lower NADPH oxidase activity in the kidney of RAP-treated mice compared to NaCl-treated mice suggests limited ROS production. RAP-treated SS mice had decreased NF-κB protein expression and activation as well as reduced TNF-α mRNA expression in the kidney. Glomerular area, interstitial fibrosis, tubular iron deposits, and KIM-1 protein expression were significantly reduced after RAP treatment. In conclusion, this study provides evidence supporting the pathogenic role of RAGE in kidney injuries in sickle cell mice.

## Introduction

Sickle cell disease (SCD) is one of the most common severe monogenic disorders worldwide. Mutated intra-erythrocytic hemoglobin S results from the substitution of valine for glutamic acid on the sixth codon of the β-globin gene (HBB) and leads to the formation of sickle-shaped red blood cells (RBCs) (Ballas and Mohandas, 1996). The homozygous disease is characterized by increased RBC fragility, decreased RBC deformability, and increased endothelial adhesion, which promote chronic hemolytic anemia and painful vaso-occlusive crises (VOC) (Rees et al., 2010; Connes et al., 2018). An imbalanced redox state and chronic inflammation also participate in the development of vasculopathy and multiple organ damage (Wood et al., 2008; Sparkenbaugh and Pawlinski, 2013; Conran and Belcher, 2018; van Beers and van Wijk, 2018). Due to its high rate of oxygen consumption and functional features, the kidney is particularly vulnerable in SCD patients. It has been estimated that 16–18% of overall mortality in patients with SCD is attributed to chronic renal failure (Platt et al., 1994). Renal manifestations of the disease include altered renal hemodynamics, renal and glomerular enlargement, and tubular deposits of iron that ultimately contribute to the development of chronic kidney disease (Nath and Hebbel, 2015).

Under oxidative conditions, advanced glycation end products (AGEs) are generated by non-enzymatic glycation and oxidation of proteins and lipids in the Maillard reaction (Singh et al., 2001). Beyond their valuable role as well-established markers of oxidative stress (Genuth et al., 2005; Koyama et al., 2007; Meerwaldt et al., 2008), it has been demonstrated that AGEs contribute to the pathophysiology of organ complications in diabetes mellitus and other chronic inflammatory diseases (Miyata et al., 1998; Huebschmann et al., 2006; Guo et al., 2012), partially through oxidative stress mechanisms/pathways (Genuth et al., 2005; Koyama et al., 2007; Meerwaldt et al., 2008). The accumulation of AGEs has been shown to participate in renal filtration alteration and glomerulopathy (Ahmed, 2005; Tan et al., 2007). The underlying molecular mechanisms involve enhanced production of pro-inflammatory cytokines, adhesion molecules, and oxidants following the activation of AGEs receptors (RAGEs) (Rojas et al., 2000; Ahmed, 2005; Goldin et al., 2006).

Although numerous SCD-related kidney complications are consistent with tissue damage induced by RAGE activation, such as albuminuria (Wendt et al., 2003), focal segmental glomerulosclerosis (Tanji et al., 2000; Wendt et al., 2003), and fibrosis (Cooper, 2004), the possible role of this receptor in the pathogenesis of SCD has been poorly investigated. To date, only two studies have reported increased plasma AGEs concentrations in children and adults with homozygous SCD at steady state with no further increase during VOC (Somjee et al., 2005; Nur et al., 2010). More recently, a third study reported increased level of AGEs in the skin of SCD patients compared to controls but the authors found no association with the clinical status of the patients (Kashyap et al., 2018). To test the hypothesis that RAGE may contribute to the development of kidney damage in SCD, we investigated the effects of RAGE inhibition on the kidney of a transgenic mouse model of SCD (Townes) expressing exclusively human sickle hemoglobin. Histological sections of the kidney, pro-inflammatory molecule expression, oxidative stress markers, and hematological parameters were analyzed in SCD mice treated with a specific antagonist peptide of RAGE.

## Materials and Methods

### Animals

We have established a colony of Townes sickle mice in our laboratory, originally purchased from the Jackson Laboratory (Bar Harbor, ME, USA). Mouse genotypes were confirmed by PCR. Townes mice have both human α- and β-globin genes knocked into the mouse locus, allowing the generation of littermates AA (healthy controls) and SS (homozygous SCD) mice (Wu et al., 2006). A total of 44 mice (21 females and 23 males) aged 8–9 weeks were used and maintained on a 12-h light–dark cycle with food and water *ad libitum*. The guidelines from the French Ministry of Agriculture for experimental procedures and the Institute for Laboratory Animal Research (National Academy of Sciences, USA) were followed and the protocol was approved by the regional animal care committee (#DR2013-46, Rhône-Alpes, France).

### Experimental Design

To determine the role of RAGE in SCD pathophysiology, RAGE antagonist peptide (RAP; 5 mg kg^−1^, #553031, Merck Millipore, Molsheim, France) was administered in 8- to 9-week-old AA and SS mice *via* intraperitoneal (IP) injection, 5 days per week for 3 weeks, as previously proposed (Arumugam et al., 2012). Saline solution (NaCl 0.9%) IP injection was used as a control.

### Tissue Sampling

The day after the last injection, mice were anesthetized with an IP injection of pentobarbital (50 mg/kg, Dolethal®, Vétoquinol, Lure, France) and blood was collected by a retro-orbital venipuncture into EDTA tubes for hematological analysis. Mice were euthanatized by exsanguination with a 0.9% NaCl transcardial perfusion for 70 s. One kidney was collected and immediately frozen in liquid nitrogen for oxidative stress and qRT-PCR analyses. The second kidney was conditioned for histology (*vide infra*).

### Hematology

An ABX Micros 60 automat (Horiba, Montpellier, France) was used for the following hematological measurements: hematocrit (Hct); red blood cell (RBC) count; hemoglobin concentration; mean corpuscular volume (MCV); RBC distribution width (RDW); mean corpuscular hemoglobin concentration (MCHC); mean corpuscular hemoglobin (MCH); white blood cell (WBC) count; lymphocyte, monocyte, and granulocyte counts. The percentage of reticulocytes and sickle cells was blindly assessed on smears stained with brilliant cresyl blue (860867, Sigma-Aldrich, St-Louis, MO, USA) by two investigators under a light microscope (BX43 Microscope, Olympus, Tokyo, Japan).

### qRT-PCR for Cytokines mRNA Expression

Total mRNA from kidney was isolated using Tri Reagent LS (Euromedex, Souffelweyersheim, France) according to the manufacturer’s instructions, purified with DNase I (EN0525, ThermoFisher scientific, Waltham, MA, USA), and concentrated at 80 ng.μl^−1^. One thousand nanograms per sample of total mRNA were reverse transcribed to cDNA with the reverse transcriptase RNase Hminus (Promega, Madison, WI, USA) using oligo (T)15 (Eurogentec, Seraing, Belgium). RT calibration was done in the presence of 80 pg. of a synthetic external and non-homologous poly(A) Standard RNA (SmRNA) used to normalize the reverse transcription of mRNAs of biological samples (Morales and Bezin, patent WO2004.092414). Real-time qPCR analysis was performed on a Rotor-Gene Q system (Qiagen, Venlo, Netherlands) by using the Rotor-Gene SYBR® green PCR kit (Qiagen, Venlo, Netherlands). The thermal profiles consisted of 15 min at 95°C for denaturing followed by 45 cycles of amplifications (15 s at 94°C for denaturation, 30 s at 58°C for annealing and, 6 s at 72°C for extension). Results obtained for the targeted mRNAs were normalized against the SmRNA. The primer pair used was: *Tumor necrosis factor-α* (TNF-α; M13049.1) forward 5′ CTG TAG CCC ACG TCG TAG C 3′, reverse 5′ TTG AGA TCC ATG CCG TTG 3′ (97 bp), *Interleukine-1*β (IL-1β; NM 008361.3) forward 5′ TTG ACG GAC CCC AAA AGA T 3′, reverse 5′ AGC TGG ATG CTC TCA TCA GG 3′ (73 bp); Interleukine-6 (IL-6; M24221) forward 5′ GCT ACC AAA CTG GAT ATA ATC AGG A 3′, reverse 5′ CCA GGT AGC TAT GGT ACT CCA GAA 3′ (78 bp); *Vascular cell adhesion molecule-1* (VCAM-1; NM 011693.2) forward 5^′^ TGG TGA AAT GGA ATC TGA ACC 3^′^, reverse 5^′^ CCC AGA TGG TGG TTT CCT T 3^′^ (86 bp).

### Oxidative Stress and Antioxidant Assessment

Kidney was homogenized (10%, w/v) in PBS 1X + EDTA 0.5 mM in ice. After centrifugation at 12,000 g for 10 min at 4°C, the supernatant was collected for measurement of oxidative stress markers. Homogenate aliquots were stored at −80°C. Protein concentrations were determined using the BCA protein assays Kit (Novagen, Darmstadt, Germany) in accordance with the manufacturer’s instructions. All of the chemicals used for oxidative stress measurements were purchased from Sigma-Aldrich (St-Louis, MO, USA) and spectrophotometric measurements were performed on TECAN Infinite 2000 plate reader (Männedorf, Switzerland). Results were standardized per mg of total protein. Glutathione peroxidase (GPx) activity was determined by the modified method of Paglia and Valentine (Paglia and Valentine, 1967). GPx activity was determined by measuring the rate of NADPH extinction after addition of glutathione reductase, reduced glutathione and NADPH using hydrogen peroxide (H_2_O_2_) as substrate as previously described (Charrin et al., 2015). NADPH oxidase activity was quantified as the formation rate of formazan blue from nitroblue tetrazolium and the superoxide radicals produced by NADPH oxidase in the presence of NADPH.

### Histology

The kidneys were harvested and fixed in a 4% paraformaldehyde (Sigma-Aldrich, St Louis, MO, USA) in a 0.1 M phosphate buffer solution for 2 h. They were then incubated in 25% sucrose (Sigma-Aldrich, St Louis, MO, USA) for 24 h for cryopreservation and gently frozen in −40°C isopentane (VWR, West Chester, PA, USA) before storage at −80°C. Seven-micrometer sections were cut and stained with hematoxylin–eosin, Masson’s trichrome, and Perl’s Blue. All observations in light microscopy were performed using a light microscope Olympus BX43 (Olympus Corporation, Tokyo, Japan), images were captured with a video camera SC30 (Olympus Corporation, Tokyo, Japan) coupled to an image analysis system (AnalySIS® getIT! 5.1; Olympus Soft Imaging Solutions GmbH, Münster, Germany). The area of 50 glomeruli per mouse was measured using Image J.

### Immunostaining

Briefly, antigen retrieval was performed by immersing frozen sections in 0.01 M citrate buffer (pH 6.0), at 95°C for 25 min. Slides were then incubated in blocking solution (TBS + 3% donkey serum) at room temperature for 1 h 30 min. Endogenous biotin and peroxidase activity were blocked before staining, by using commercial avidin/biotin and peroxidase kits, respectively (Vector Lab, Burlingame, CA, USA). Slides were incubated overnight at 4°C with the following primary antibodies: rabbit polyclonal anti-NF-κB p65 (sc-372, dilution 1:200, Santa Cruz Biotechnology, CA), mouse monoclonal anti-phosphorylated NF-κB p65 Ser536 (sc-136,548, dilution 1:200, Santa Cruz Biotechnology), or rat monoclonal anti-KIM1 (sc-53,769, dilution 1:50, Santa Cruz Biotechnology). After washing, sections were then incubated with a biotinylated donkey anti-rabbit (711-065-152, dilution 1:2,000; Jackson Immuno-Research, Suffolk, UK), donkey anti-mouse (715-065-150, dilution 1:5,000; Jackson Immuno-Research), or donkey anti-rat antibody (712-065-153, dilution 1:2,000; Jackson Immuno-Research). Exposure was performed with the avidin-biotin enzyme complex (Vectastain Elite ABC standard peroxidase Kit; Vector Lab, Burlingame, CA, USA) and the substrate 3,3′-diaminobenzidine (DAB Peroxidase Substrate Kit; Vector Lab, Burlingame, CA, USA). ImageJ® software with the “Immunoratio” plugin was used to semi-quantify NF-κB p65, phosphorylated NF-κB p65 Ser536, and KIM-1 expression in 30–50 randomly selected cortical areas. This score was measured by determining the total tissue area on the original picture while the DAB-positive area was defined using ImageJ’s automatic threshold on the DAB component, obtained as previously described (Tuominen et al., 2010).

### Statistics

Statistical analyses were performed using Statistica Software (Tulsa, OK, USA). All variables were tested for normality and variance homogeneity. Data were analyzed using two-way ANOVA followed by planned comparisons or Student’s *t*-test when appropriate. A “*p*-value” inferior to 0.05 was considered statistically significant. The data were expressed as means ± SD.

## Results

### Receptor for Advanced Glycation End Product Blockade Blunts Kidney Damage in SS mice

**Figure 1:**
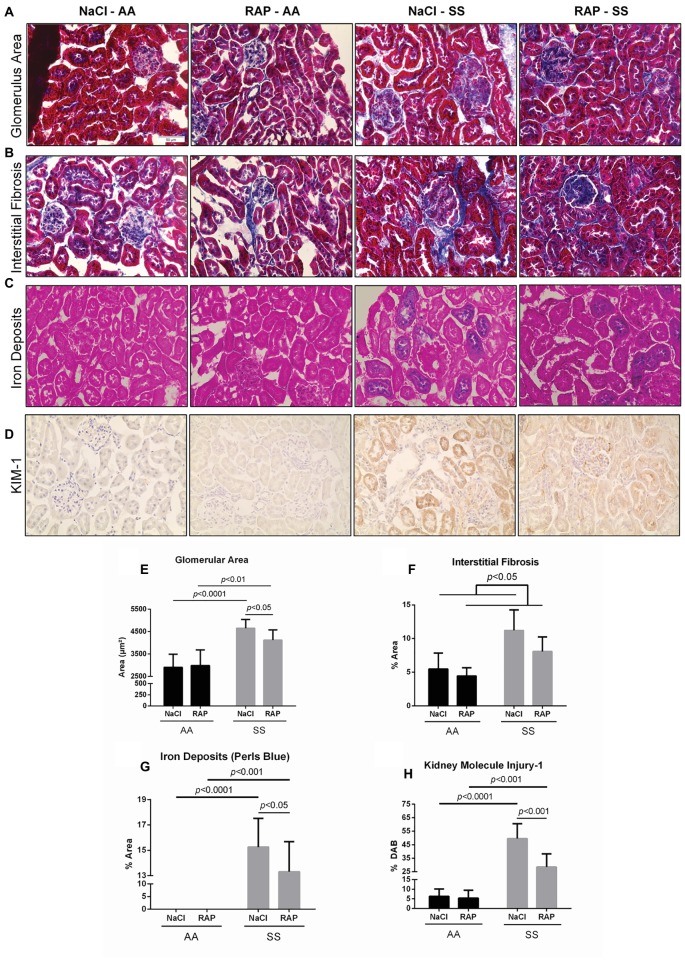
Histopathological analysis of changes in morphology of 11- to 12-week-old AA and SS mice kidneys after 3 weeks of treatment with RAGE antagonist peptide. Representative images with Masson’s trichrome staining **(A,B)** for determining glomerular area and interstitial fibrosis and Perl’s Blue staining **(C)** for determining iron deposits **(D)** representative images of KIM-1 stained kidney sections. Magnification: ×400. Quantification of glomerular area **(E)**, interstitial fibrosis **(F)**, tubular iron deposits **(G)**, and KIM-1 expression **(H)**. Values are means ± SD. NaCl-AA (*n* = 6; three females and three males), RAP-AA (*n* = 6; four females and two males), NaCl-SS (*n* = 7; three females and four males), RAP-SS (*n* = 7; three females and four males). Scale bar = 50 μm.

Renal histology as assessed by Masson’s trichrome staining revealed glomerular hypertrophy demonstrated by higher glomerular area (*p* < 0.05; Figures 1A,E) and higher interstitial fibrosis (*p* < 0.05; Figures 1B,F) in SS compared to AA mice. Remarkably, RAGE inhibition lowered the glomerular area in SS mice (*p* < 0.05; Figures 1A,E). In addition, an overall treatment effect on interstitial fibrosis was detectable in the RAP-treated group compared with the NaCl-treated group (*p* < 0.05; Figures 1B,F). Marked accumulation of iron deposits was observed on kidney sections of SS mice stained by Perl’s Blue compared to their AA littermates (*p* < 0.01; Figures 1C,G) but the number of iron-positive tubules was significantly decreased in RAP-SS compared to NaCl-SS mice (Figures 1C,G). Finally, while tubular and glomerular accumulation of KIM-1 was exacerbated in SS compared to AA mice, RAGE blockade blunted KIM-1 immunostaining in SS when compared to NaCl-SS mice (Figures 1D,H). Results were similar between male and female mice (data not shown).

### Receptor for Advanced Glycation End Product Inhibition Modulates NAPDH Oxidase and Glutathione Peroxidase Activity in SS mice

**Figure 2:**
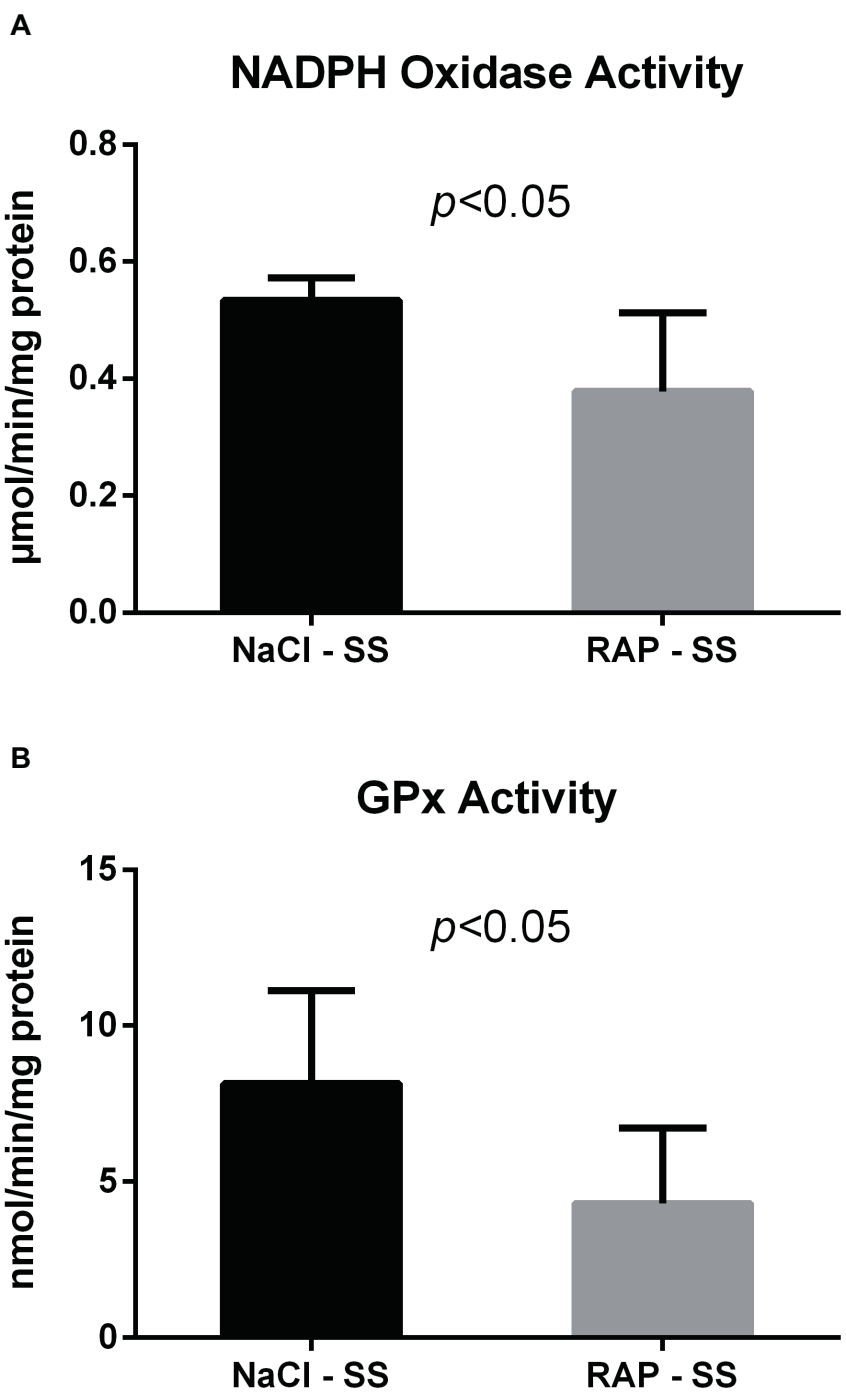
Oxidative stress marker **(A)** and antioxidant enzyme **(B)** activities after RAGE antagonist peptide treatment in the kidney of AA and SS mice. Values are means ± SD. GPx, Glutathione Peroxidase. NaCl-SS (*n* = 7; three females and four males), RAP-SS (*n* = 7; three females and four males).

We next examined whether NADPH oxidase – which can be activated by RAGE (Wautier et al., 2001) – was modulated by RAGE antagonist peptide (RAP) treatment in the kidney of sickle cell mice. Both NADPH oxidase and GPx activities were reduced in the kidney of RAP-SS compared to NaCl-SS mice (*p* < 0.05; Figure 2).

### Blockade With Receptor for Advanced Glycation End Product Antagonist Peptide Decreases Kidney Inflammation

**Figure 3:**
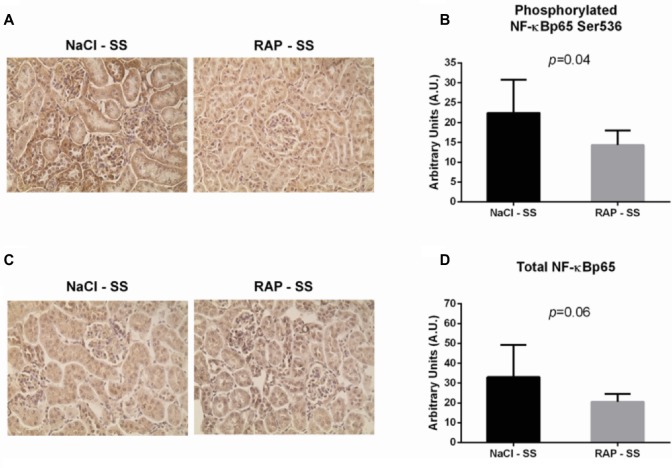
Effect of RAP treatment on protein and mRNA expression of molecules acting downstream of the RAGE signaling pathway. Kidney sections from NaCl-SS and RAP-SS mice were subjected to immunohistochemistry using anti-NF-κBp65 IgG **(A)** and anti-phosphorylated NF-κBp65 Ser 536 IgG **(B)**, Magnification: ×400. Staining score **(C,D)** was determined using ImageJ plugin “Immunoratio”. Values are means ± SD. NaCl-SS (*n* = 7; three females and four males), RAP-SS (*n* = 7; three females and four males).

To further understand the role of RAGE on kidney pathophysiology in sickle cell mice, we assessed NF-κB protein expression and TNF-α genic expression, key pro-inflammatory molecule acting downstream of the RAGE pathway. After 3 weeks of treatment, phosphorylated NF-κBp65 Ser536 staining was lower (*p* < 0.05) in RAP-SS compared to NaCl-SS mice (Figures 3A,B). RAP treatment did not significantly change (*p* = 0.06) total NF-κBp65 expression on SS mice kidney sections in comparison with their NaCl-treated littermates (Figures 3C,D). Finally, TNF-α mRNA expression was five times greater in the kidney of NaCl-SS (Table 1) than in NaCl-AA mice. In contrast, TNF-α mRNA was significantly reduced in RAP-SS kidney (*p* < 0.05; Table 1) compared with that of NaCl-SS. The seemingly present increase in TNF-α mRNA after RAP is not significant in the AA group. No significant difference was detected for IL-1, IL-6, and VCAM-1 mRNA expression in SS group after RAP treatment (Table 1).

**Table 1:** Renal mRNA expression of inflammatory and adhesion cell markers in NaCl- or RAP-treated AA and SS mice.

	NaCl-AA	RAP-AA	NaCl-SS	RAP-SS
TNF-α (No. of copies)	50.1 ± 49.5	110.4 ± 88.7	247.3 ± 187.2^*^	132.9 ± 105.4^$^
IL-1β (No. of copies)	261.0 ± 173.7	232.8 ± 89.6	578.9 ± 254.9	602.2 ± 299.9^†^
IL-6 (No. of copies)	93.3 ± 97.8	36.0 ± 25.6	113.6 ± 59.8	185.1 ± 191.0
VCAM-1 (No. of copies)	4905.5 ± 4601.6	7716.9 ± 2556.3	13790.8 ± 6839.5	25046.9 ± 19009.7^†^

IL-1β, Interleukin-1β; IL-6, Interleukin-6; VCAM-1, Vascular Cell Adhesion Molecule-1.

* p < 0.01 vs. NaCl-AA;

† p < 0.05 vs. RAP-AA;

$ *p < 0.05 vs. NaCl-SS*.

*NaCl-AA (*n* = 6; three females and three males), RAP-AA (*n* = 6; four females and two males), NaCl-SS (*n* = 7; three females and four males), RAP-SS (*n* = 7; three females and four males)*.

### Receptor for Advanced Glycation End Product Inhibition Limits Anemia

**Figure 4:**
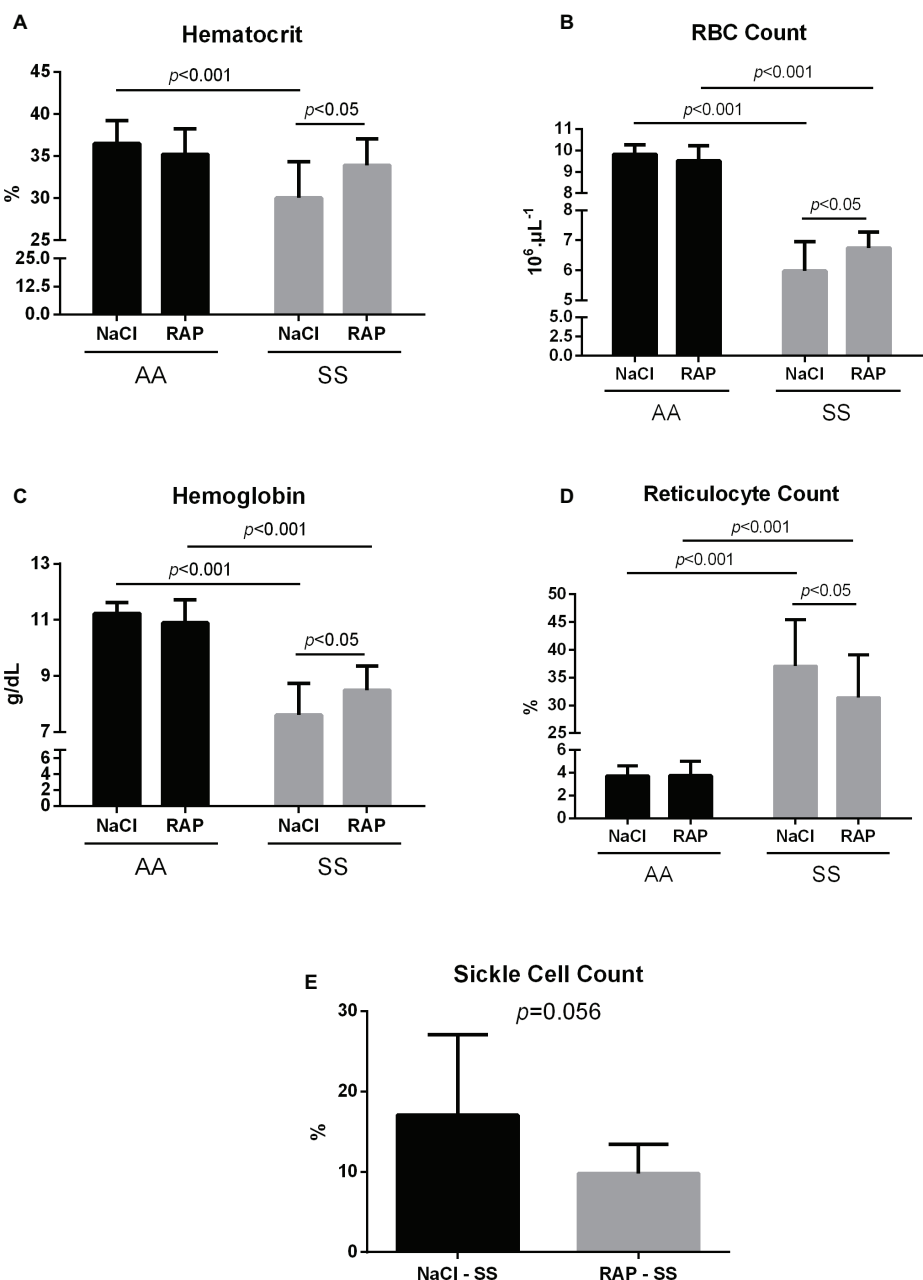
Mean hematocrit **(A)**, red blood cell count **(B)**, hemoglobin levels **(C)**, reticulocyte count **(D)**, and sickle cell count **(E)** after treatment in AA and SS mice. Values are means ± SD. RBC: Red Blood Cell. NaCl-AA (*n* = 13; five females and eight males), RAP-AA (*n* = 12; seven females and five males), NaCl-SS (*n* = 10; five females and five males), RAP-SS (*n* = 9; four females and five males).

Hematological changes are detailed in Figure 4. MCV, RDW, MCH, WBCs, and reticulocyte count were significantly higher in the SS group while MCHC, hematocrit, RBCs, and hemoglobin level were lower in SS mice than in their AA littermates (Table 2, Figure 4). In RAP-treated SS mice, there was no treatment effect on WBCs (Table 2). However, RBC count and hemoglobin level were increased (*p* < 0.05; Figures 4A–C). Sickle cell percentage as well as reticulocyte count decreased in RAP-treated SS compared to NaCl-SS mice (*p* < 0.05; Figures 4D,E).

**Table 2:** Hematological indices in NaCl- or RAP-treated AA and SS mice.

	NaCl-AA	RAP-AA	NaCl-SS	RAP-SS
MCV (fl)	37.2 ± 2.5	37.2 ± 2.0	50.6 ± 4.1^*^	50.2 ± 2.2^†^
RDW (%)	15.3 ± 0.6	15.3 ± 1.1	23.0 ± 2.1^*^	22.4 ± 1.5^†^
MCHC (g dl^−1^)	31.3 ± 0.8	30.9 ± 1.7	25.3 ± 0.9^*^	25.2 ± 0.8^†^
MCH (pg)	12.0 ± 0.9	11.5 ± 0.5	12.8 ± 0.9^*^	12.7 ± 0.7^†^
WBC (10^3^ μl^−1^)	4.8 ± 1.2	4.2 ± 1.9	43.2 ± 6.7^*^	47.7 ± 11.0^†^
Lymphocytes (10^3^ μl^−1^)	3.6 ± 0.8	3.3 ± 1.5	38.1 ± 6.1^*^	42.9 ± 9.3^†^
Monocytes (10^3^ μl^−1^)	0.4 ± 0.2	0.3 ± 0.2	2.2 ± 0.8^*^	1.9 ± 0.8^†^
Granulocytes (10^3^ μl^−1^)	0.9 ± 0.4	0.6 ± 0.3	2.9 ± 1.4^*^	2.9 ± 1.9^†^

*Values are presented as means ± SD. MCV, Mean corpuscular volume; RDW, Red blood cell distribution width; MCHC, Mean corpuscular hemoglobin concentration; MCH, Mean corpuscular hemoglobin; WBC, White blood cell*.

* p < 0.001 vs. NaCl-AA;

† *p < 0.001 vs. RAP-AA*.

*NaCl-AA (*n* = 13; five females and eight males), RAP-AA (*n* = 12; seven females and five males), NaCl-SS (*n* = 10; five females and five males), RAP-SS (*n* = 9; four females and five males)*.

## Discussion

The current study aimed to investigate the effect of RAGE inhibition on markers of kidney damage as well as on markers of oxidative stress and inflammation in the kidney of homozygous sickle mice. In support of our hypothesis, the results of the present study demonstrated for the first time that a RAGE blockade (1) dampened kidney damage, as evidenced by reduced glomerular hypertrophy, interstitial fibrosis, iron deposition, and KIM-1 protein expression in SS mice; (2) reduced the activation of both NADPH oxidase and NF-κBp65 acting downstream of the AGE/RAGE signaling pathway; (3) increased hematocrit, RBC count, and hemoglobin level, and decreased reticulocyte count and sickle cell count in SS mice.

While SS mice displayed common renal manifestations of SCD, i.e., glomerular hypertrophy (Elfenbein et al., 1974; Bhathena and Sondheimer, 1991), interstitial fibrosis (Walker et al., 1971; Alhwiesh, 2014), iron overload (Walker et al., 1971; Buckalew and Someren, 1974), and KIM-1 overexpression (Sundaram et al., 2011; Hamideh et al., 2014) – a specific marker of tubular injuries – RAP treatment minimized kidney injuries in these mice. Our findings are in agreement with those of a previous study performed in diabetic mice where administration of soluble RAGE reduced glomerular area (Wendt et al., 2003). In nephropathies, it was reported that glomerular hypertrophy results from podocyte hypertrophy and extracellular matrix (ECM) accumulation (Li et al., 2007), and RAGE activation was shown to contribute to both of these pathological changes (Liebisch et al., 2014; Zhao et al., 2014). Through the inhibition of the expression of the protein NIPP1, AGE/RAGE interaction induced cell cycle arrest and concomitant podocyte hypertrophy. Interestingly, the activation of this pathway was NF-κB/TNF-α dependent (Liebisch et al., 2014). Similarly, ECM accumulation has been shown to be mediated by the AGE/RAGE axis and the NF-κB signaling pathway, which are involved in ECM synthesis and myofibroblast differentiation (Zhao et al., 2014). Thus, both glomerular hypertrophy and interstitial fibrosis – which also results from ECM accumulation in the interstitium and myofibroblast differentiation (Farris and Colvin, 2012) – may be sustained by AGE/RAGE/NF-κB signaling in sickle cell mice. Nevertheless, additional quantitative measurements on the expression of fibrosis markers (i.e., Col1α1, α-SMA, Vimentin, Fibronectin) are required to confirm this assumption. Tubular iron deposition is a common feature of SCD, as free plasma HbS pass through the glomerular filtration barrier and are incorporated into renal tubules (Nath and Hebbel, 2015). As iron deposits in the cortex of SCD patients were associated with intravascular hemolysis, one could hypothesize that the decrease in tubular iron deposits measured in our SS mice might be related to the reduced anemia we observed after RAP treatment. Interestingly, we observed an increase in hematocrit, RBC count, and hemoglobin level and a decrease in reticulocyte count in RAP-treated sickle mice that could suggest that RAGE may play a significant role in anemia. This finding could most likely be explained by decreased hemolysis rather than increased erythropoietic process, as a previous study reported a role of AGEs in the pathophysiology of chronic hemolysis-associated organ complications in SCD (Nur et al., 2010). Nevertheless, further studies are required to elucidate the role of the AGE/RAGE pathway on hemolytic processes. Finally, KIM-1 is commonly used to assess acute tubular injury as it is expressed specifically on damaged tubules but is undetectable in healthy ones (van Timmeren et al., 2007). In a recent study, urinary KIM-1 levels were reduced in diabetic RAGE-KO mice compared to diabetic wildtype mice (Thallas-Bonke et al., 2013), which is consistent with the results in the present study. Furthermore, KIM-1 has been shown to be associated with renal fibrosis and inflammation (Humphreys et al., 2013), which further supports the implication of the RAGE signaling pathway in SCD-related kidney disease.

Considerable evidence demonstrates increased oxidative stress in sickle cell disease (Chirico and Pialoux, 2012; Charrin et al., 2016). The primary mechanism by which RAGE generates oxidative stress is *via* the activation of NADPH oxidase (Gao et al., 2008). The downward RAP treatment effect on renal NADPH oxidase activity could suggest blunted basal oxidative stress in mice treated with RAGE antagonist that may explain the lower GPx activity in RAP-SS compared to vehicle-SS mice. This hypothesis is supported by previous work showing reduced NADPH oxidase activity and nitrotyrosine levels in a glomerulosclerosis mouse model either knocked-out for RAGE or treated with soluble RAGEs (sRAGEs) (Guo et al., 2008). In these mice, RAGE blockade also improved albuminuria and limited glomerular sclerosis. Additionally, other studies reported decreased intracellular reactive oxygen species (ROS) after inhibition of RAGE with either RAGE-shRNA in renal fibroblasts (Chen et al., 2010) or RAGE antibody in renal mesangial cells (Ide et al., 2010). Collectively, our data strongly suggest that RAGE blockade is likely to ameliorate oxidative stress status in the kidney of sickle mice and may further support the hypothesis of a reduced anemia after RAP treatment.

As inflammation plays a key role in the pathophysiology of SCD (Hoppe, 2014) and is potentiated by RAGE activation (Goldin et al., 2006), we assessed protein expression of a key inflammatory molecule, i.e., NF-κBp65, and one of its target genes (i.e., TNF-α) at the mRNA level (Figure 3, Table 1). In the kidney of our vehicle-SS Townes mice, the high gene expression of pro-inflammatory cytokine TNF-α strengthens the assumption of a renal pro-inflammatory state in SCD (Akohoue et al., 2007; Hebbel et al., 2009; Krishnan et al., 2010). Interestingly, RAP treatment dampened phosphorylated NF-κBp65 expression in our SS mice. Consistent with this, it was reported that blockade of RAGE with either soluble RAGE or FPS-ZM1 suppressed NF-κB pathway in a murine model of systolic overload-induced heart failure (Liu et al., 2016). In addition, Flyvbjerg et al. reported a decrease in renal NF-κB expression along with an overall improvement of kidney function after treatment with RAGE antibody in obese Type 2 diabetic mice (Flyvbjerg et al., 2004). Thus, in the present study, inhibition of NF-κB in RAP-SS mice could explain the reduction of TNF-α mRNA levels to close to the levels observed in healthy mice. In line with this observation, recent studies showed decreased cardiac TNF-α mRNA expression in a mouse model of inflammatory heart disease knocked-out for RAGE (Bangert et al., 2016) and lower hepatic TNF-α mRNA in RAGE−/− mice after ischemia/reperfusion injury (Zeng et al., 2009). A similar drop in aortic TNF-α mRNA occurred in sinoaortic denervated rats treated with sRAGEs, acting as a decoy for RAGE (Wu et al., 2013). In this context, our data suggest that RAGE inhibition could weaken pro-inflammatory processes in the kidney of sickle cell mice.

In conclusion, our data suggest that specific inhibition of RAGE could blunt anemia-related markers. Both RAP-mediated reduced oxidative stress markers and decreased pro-inflammatory molecule expression might take part in reducing the hemolytic process as well as the glomerular hypertrophy, interstitial fibrosis, and iron deposits in the kidney of sickle cell mice. Although further studies are warranted to elucidate the role of RAGE on kidney function in sickle cell disease, our data demonstrate that this receptor seems to be an important pathogenic factor in the development of renal changes in SCD mice. Only one clinical grade antagonist of RAGE (Azeliragon: TTP488) has been tested in Alzheimer’s disease patients only, in Phase I, II (Burstein et al., 2014, BMC Neurobiol.), and III clinical trials (NCT02080364, Clinicaltrial.gov). Results of Phase III are not available at this time.

### Limitations

Our study has some limitations. The study was primarily designed to investigate acute effects of RAGE inhibition on sickle cell mice. Therefore, no functional nor mechanistic experiments were performed and thus no definitive conclusions about kidney function can be drawn.

## Ethics Statement

Comité d’Ethique en Expérimentation Animale CEEA-55 Project 2017020817227030_v1.

## Author Contributions

EC and CM participated in the design of the study. EC, CF, and AS performed the experiments. EC, CF, SS, VP, PJ, PC, and CM wrote the manuscript.

### Conflict of Interest Statement

The authors declare that the research was conducted in the absence of any commercial or financial relationships that could be construed as a potential conflict of interest.
